# Barriers and enablers of women carers from culturally and linguistically diverse communities participating in physical activities

**DOI:** 10.3389/fspor.2024.1444025

**Published:** 2024-08-29

**Authors:** Pam Kappelides

**Affiliations:** Human Services and Sport, School of Allied Health, La Trobe University, Melbourne, VIC, Australia

**Keywords:** CALD women, phenomenological evaluation, physical activity, wellbeing, inclusivity

## Abstract

**Introduction:**

This research article presents a phenomenological evaluation of an organization working within disability and with CALD communities of a project that was supporting women carers who are newly arrived to Australia to be happier and healthier. The initiative aimed to alleviate social isolation, enhance wellbeing, and boost confidence among women from Culturally and Linguistically Diverse (CALD) backgrounds in Metropolitan Melbourne, Victoria.

**Methods:**

Through a qualitative exploration of the lived experiences of participants, findings were categorized into enablers and barriers, shedding light on the outcomes of physical activity participation for these women.

**Results and discussion:**

Enablers included improvements in health, skill development, achievement, and enhanced social interaction and support, while barriers encompassed challenges related to access, socio-cultural factors, resource availability, interpersonal dynamics, and physiological constraints. Based on these findings, recommendations are proposed to empower CALD community members in the co-development and co-delivery of future programs, fostering sustainability and community-driven engagement.

## Introduction

As societies evolve, the importance of inclusivity and diversity becomes increasingly evident (1, 2) necessitating tailored interventions to address the specific needs of marginalized communities in sport and recreation programs.

The increasing diversity of populations in many countries has brought attention to the unique challenges faced by culturally and linguistically diverse (CALD) communities, particularly in the realm of caregiving. CALD carers, who provide unpaid care for family members or friends, often navigate additional barriers compared to their non-CALD counterparts.

Physical activity (PA) is well-documented for its numerous health benefits, including the reduction of chronic disease risk, improvement in mental health, and enhancement of overall physical well-being (3, 4). Regular PA strengthens the heart and improves circulation, which can help prevent cardiovascular diseases such as heart attacks and strokes (5). This is especially important for carers, who may have an increased risk of heart disease due to stress and sedentary lifestyles. Engaging in activities such as walking, strength training, and flexibility exercises helps maintain muscle strength, bone density and joint flexibility. This is crucial for carers who may need to perform physical tasks, such as lifting or assisting their care recipients. Research has shown that exercise can alleviate symptoms of depression and anxiety by increasing serotonin and dopamine levels in the brain (6). This can help carers maintain a more positive outlook and emotional balance, essential for coping with the demands of caregiving. We also need to understand how engaging in group physical activities, such as walking clubs or exercise classes, provides opportunities for social interaction and support. Which can help alleviate feelings of isolation and loneliness that many CALD carers may experience (7).

For carers, maintaining a regular routine of PA is particularly important due to the physically and emotionally demanding nature of caregiving. However, CALD carers often experience lower levels of PA due to their unique circumstances, which can exacerbate health disparities and negatively impact their caregiving capabilities.

An organization that works specifically with carers from CALD communities within a disability context recognized a need to foster and engage CALD women in a project that aimed to foster holistic wellbeing. By focusing on social isolation reduction, wellbeing enhancement, and confidence building, the project sought to empower participants to actively engage in activities beyond the project's duration, thereby promoting sustained community involvement. The following research questions were examined:

1. Do women carers from CALD backgrounds participate in PA?
2. What are the enablers to participation in PA?
3. What are the barriers to participation in PA?
4. What strategies have been effective in promoting PA among CALD communities?

The methodological approach employed in this evaluation reflects a commitment to capturing the nuanced experiences of CALD women across various ethnic backgrounds. Through a phenomenological lens, the research delved into the subjective realities of participants, acknowledging the diversity of their lived experiences. The inclusion of women from Afghan, Chinese, Ethiopian, Filipino, Greek, Indian, Karen, Karenin, Nigerian, South Sudanese, Sri Lankan, Syrian, and Vietnamese backgrounds ensures a comprehensive understanding of the intersectional challenges and opportunities inherent within CALD communities.

### Organisation context

This research study was conducted for a community-based organization that serves individuals with disabilities from CALD backgrounds. This organization, hereafter will be referred to as Organisation 1 (O1). The organization provides a range of services including advocacy, information dissemination, referrals, education, training, and consultancy. O1 primarily assists individuals with disabilities from CALD backgrounds as well as their caregivers.

One notable service offered by O1 is the facilitation of bilingual support groups. These groups provide a platform for individuals with disabilities and their caregivers from CALD backgrounds to gather regularly for social support, information sharing, and educational purposes. Additionally, O1 serves as a valuable resource for service providers, community organizations, and government departments, functioning as a conduit for individuals from CALD backgrounds with disabilities and carers to access essential services.

Utilizing O1's services yields numerous benefits for individuals with disabilities and carers from CALD backgrounds. They gain a platform to voice their concerns and advocate for their rights, establish support networks within their ethnic communities and the broader society, and receive education and information about available services and resources. Furthermore, service providers, community organizations, and government departments benefit from O1's expertise and guidance, receiving assistance in tailoring programs and services to meet the needs of CALD communities.

### Theoretical background

Sport, PA and recreation are integral components of a healthy lifestyle, offering numerous physical, social and psychological benefits. However, despite efforts to promote inclusivity and diversity within these domains, individuals from CALD backgrounds, including newly arrived migrants, continue to face barriers to participation (8).

Physical inactivity is a global public health issue, particularly prevalent among women in CALD populations. These women often face unique barriers to PA, including cultural norms, socioeconomic status, and access to safe and appropriate facilities. Understanding these patterns and barriers can help in designing targeted interventions to promote PA (2, 9, 10).

Globally, research (1, 11, 12) has shown similar patterns of lower participation rates in sport, PA and recreation among CALD communities. Studies from various countries, including the United States, Canada, and the United Kingdom, highlight common barriers such as language barriers, cultural norms, socioeconomic disparities, and discrimination (13, 14). These findings underscore the universality of the challenges faced by CALD communities in accessing and engaging in PA. In Australia, in 2016, the Australian Sport Commission released the Aus Play survey. The survey provides data for the sport and recreations industry about participation rates in both organised and non-organised sport and recreation. Aus Play asks survey respondents if they speak a language other than English at home. Data released in 2016 showed that people who spoke a language other than English at home were less likely to participate in sport or PA than those who spoke only English. Adults who speak a language other than English at home are 10% less likely to participate in sport, PA or recreation activities at a rate of 3 times per week. Research also shows that CALD women born outside of Australia have reported 20% less participation in sport, PA and recreation compared to Australian born women (46.3% in CALD woman compared to 66.5% in Australian born women) (15). Children who speak a language other than English at home are 14% less likely to participate in sport, PA, or recreation at least once per year (outside of school hours) (16). With almost 50% of the Australian population either born overseas or with a parent born overseas, there is an enormous opportunity to engage more people from CALD backgrounds in sport, PA, and recreation.

CALD carers face unique challenges that can impact their physical activity levels and overall health. Research indicates that CALD carers often experience higher levels of stress and lower levels of PA compared to their non-CALD counterparts, primarily due to cultural, social and structural barriers (17). Additionally, CALD carers frequently report a lack of time as a major impediment, as caregiving responsibilities can be particularly demanding and time-consuming (9, 10).

For CALD carers, who may experience higher levels of physical and emotional stress, staying active can help manage or mitigate these health risks. PA also strengthens the immune system and improves cardiovascular health, which is vital for carers who need to maintain their health to provide care (18). Caring for a loved one can be emotionally draining and stressful, particularly for CALD carers who might also face language barriers and cultural isolation.

PA is a proven stress reliever, helping to reduce symptoms of depression and anxiety through the production of endorphins, which are natural mood lifters (18, 19). Walker et al. (20) pointed out that regular exercise also contributes to better sleep, which can be especially beneficial for carers who might struggle with disrupted sleep patterns due to their caregiving responsibilities. Physical activity can also provide crucial opportunities for social interaction and community engagement for CALD carers, who often feel isolated due to cultural and linguistic barriers. Participating in activities such as walking groups or exercise can help build networks of support, foster friendships, and provide a sense of community and belonging, which is essential for emotional resilience. Moreso, the physical demands of caregiving, such as lifting or transferring to care for someone, require physical strength, flexibility, and endurance. Regular PA helps maintain and improve these physical capabilities, ensuring that carers are less susceptible to injuries and physical strain (21).

By improving physical and mental health, PA helps enhance the overall quality of life for CALD carers. It contributes to higher energy levels, better mood, and a more positive outlook on life, all of which are important for maintaining independence and fulfilling the demanding role of a caregiver (17, 22).

Participation in sport, PA, and recreation is influenced by a range of social determinants of health, including socioeconomic status, education level, cultural norms, and access to resources. Newly arrived migrants and individuals from CALD backgrounds often face socioeconomic challenges, language barriers, and limited access to sporting facilities and programs, which can hinder their participation. Additionally, cultural norms and attitudes towards PA may differ across ethnic groups, impacting engagement levels (23). Furthermore, perceptions of discrimination or exclusion within sporting or recreation environments can deter participation and perpetuate feelings of marginalization as outlined in Spaaij et al. (24).

Also, acculturation, the process by which individuals adopt the cultural norms and practices of their new society, plays a significant role in shaping participation patterns among newly arrived migrants (25). For some, as pointed out by Khawaja, Moisuc and Ramirez (26) participation in sport and PA may facilitate social integration and a sense of belonging in their new community. However, acculturation can also pose challenges, as individuals navigate cultural differences and seek to maintain connections to their heritage while adapting to their new environment.

## Research methodology

### Participants

This research study was led by a phenomenological methodology to explore lived experiences. Twenty-six separate focus groups were undertaken to explore how women from CALD communities experienced sport, PA, and recreation programs designed and organised by O1 across various locations in Metropolitan Melbourne, Victoria. Using the phenomenological focus group approach allows for a supportive environment for individuals to share their lived experiences, which may not be feasible using alternative methods (9). The study was approved by the Human Research Ethics Committee of {left out for blind review}.

### Recruitment

We used a purposeful participant sampling method of CALD community members engaged with O1 in the sport, PA and recreation programs of tennis, yoga, gym exercise, dance, swimming and self-defence offered. Participants came from the targeted communities of Afghan, Chinese, Ethiopian, Filipino, Greek, Indian, Karen, Karenin, Nigerian, South Sudanese, Sri Lankan, Syrian and Vietnamese for which these programs were organized. The majority of women were aged 35–65. Service providers and group facilitators (many of whom were from CALD background) were also included in the focus groups. A key aspect of the focus groups was that a language translator (group facilitator) was used as English was limited for the majority of the research participants and allowed each woman to understand and feel comfortable to respond in their native language.

Each focus group allowed for each participant's interpretation of their sport, PA or recreation programs experiences that were explored though in-depth discussions. The focus groups used a semi-structured interview schedule. The objectives of the research project and based on previous literature concerning the PA and health behaviours of women from CALD populations guided the questions. Focused groups ranged from 6 to 12 participants in each, and 26 focused groups were conducted (*N* = 269 participants). Each participant attended an initial focus group when the activity first started, an end focus group once the activity ended (either 6–8-week block) with a follow up six months and then another focus group 12 months after the first focus group. Questions were asked about participants’ direct experiences in the sport, PA or recreation programs they participated in. For example: “what does sport, PA or recreation mean to you?”, “what made you choose this program?”, “what are the best things about being involved in this program?” and “have you seen any changes to yourself during or after the program?”. The questions were intentionally open and wide-ranging to enable participants to discuss all relevant experiences to, PA or recreation.

We also asked questions to uncover some of the barriers and enablers to sport, PA and recreation participation in CALD groups. Participants were asked about some reasons why they and other people from the same community might not participate in sport, PA, and recreation and also what things and/or reasons would motivate them to regularly participate in PA programs.

The service providers and group facilitators (*N* = 30) who worked directly with the woman were asked questions such as: “how do you feel your programs are benefiting the participants?”, “how do you see friendships/connections occurring in these programs?”. All the focus groups were held on-site or *via* zoom where each of the programs was delivered.

### Data collection and analysis

Group facilitators were contacted directly by the researcher to organise the semi-structured focus groups with each CALD group participating in the various sport, PA or recreation programs. Focus groups were conducted face to face and *via* zoom. Focus groups lasted between 45 min and 65 min. Prior to the focus groups, participants were provided with a participant information sheet and consent form in the various native languages of the participants (Afghan, Chinese, Ethiopian, Filipino, Greek, Indian, Karen, Karenin, Nigerian, South Sudanese, Sri Lankan, Syrian and Vietnamese) as well as English. Consent forms were emailed to the researcher *via* the group facilitators prior to the focus groups being conducted. Participants were informed about confidentiality, information about the study and about the right to withdraw from the study in the consent form and then prior to starting each focus group.

Focus groups were audio recorded and transcribed verbatim. Data were analysed using inductive data-led approach, following Braun and Clarkes (27, 28) six-phase process of reflective analysis. The analysis was conducted by two researchers, who familiarised themselves with the whole dataset by reading, re-reading and note-taking. Each of the researchers then independently coded six of the transcripts, generating inductive codes derived from the dataset, for example “increased skills” and “health benefits”. Initial codes and thoughts were discussed and agreed before the remaining transcripts were inductively coded by both researchers. Following the coding process, initial themes were generated by one of the researchers, which were discussed and disagreements between the coders were discussed and resolved at this stage. The initial themes were developed and refined into a final set of themes and sub-themes, reviewing these against the coded extracts and the overall dataset. There was ongoing discussion between the researchers throughout the analytic process in the defining and refining of the final meaningful themes from the dataset and naming them appropriately. Following the initial process of reading the transcripts, the data were imported into a NUD*IST Vivo software program (NVivo). The NVivo program was used to code the data from individual transcripts and facilitate a more comprehensive analysis of the interview. Finally, the themes, interpretations of their meaning, and supporting extracts from the data were written up for reporting.

## Results

The findings of this study have been organized into categories of enablers and positive outcomes, which include health and well-being advantages, skill development and accomplishments, social interactions and support. Additionally, the study pinpointed several barriers that frequently obstruct participation in sports, PA, and recreation.

### Enablers

#### Health and well-being advantages

A key benefit for CALD community women from participating in the O1 Program was improved health and wellbeing. Many women in the various focus groups mentioned increased physical competence, having more energy, growing motivation, confidence, knowledge and understanding to value and take responsibility to be active in their daily life. Participants said:

“*I never really did anything. I never liked sports before we started this [Yoga) but, now I really do and feel flexible and stronger in my tummy.”*

*“I got much better over the few times at tennis, I was more good at hitting the ball back because I was more confident.”*

*“First ever time doing yoga or any type of exercise even. Didn't know my bones were so tight.”*

*“I feel happy because I learned a lot of stuff about being safe [self-defence].”*

*“I am motivated now to get off the couch and do something, even if it’s walking each day.”*

*“I went to the doctor after doing Yoga program and he said my blood pressure was lower, he gave me less medication.”*

#### Skill development and accomplishments

The research identified that having programs that teach a life skill such as swimming or self-defence were well received and value-adding to the program. That is, the skills participants were developing outside of the physical outcomes was important. For example, it was the longer-term positive outcomes that they could take with them after the program was delivered. They also highlighted that having these type of survival skills was essential to living in Australia, particularly exemplified by the swimming program.

“*Before I started this program, I felt shy to go to the pool with my family because I didn’t know how to swim.”*

*“I like swimming, it’s good to learn. Before when I was going to the sea, just looking, before I get swimming lessons, I was saying “no I can’t” but now, I’m not perfect but I try to swim for myself.”*

*“I feel more confident that I protect myself when I walk home from work.”*

*“I lost 5 kilo and I think it was from working out and doing yoga 2 times per week and also doing it at home in my spare time. I'm so happy.”*

#### Social relationships

There was a strong emphasis from each focus group on friendship, relationship building and social interaction as a main contributor to their ongoing participation with the activity and group. While not all participants were always from the same ethnic or cultural background, there was an element of commonalty and mutual support that was identified by being from the same organisation, O1 and carers. Participants talked about the importance of having others to talk about personal issues outside of the activity and being part of a support network was very important.

“*I am making a lot of friends. Before I was at home with my children and depressed, it’s nice to be able to meet other lovely ladies.”*

*“I liked all the compliments that I received on my track suits and lip stick. It made me feel happy and I enjoyed the session.”*

*“We are friends here and we can talk about how we are doing, they understand, they are also carers, it helps that we all speak the same language.”*

*[The swimming session is ] “Very good, providing us the chance to socialise, exercise and relaxing time.”*

*“Before I came to this group, I didn’t know anyone or anything to do. Now I can ring any lady from here when I have a question, need help or want to talk, they are like family, community.”*

#### Support

The findings indicate that the most valuable aspects of these programs lie in O1's support to provide accessible, supportive and appropriate opportunities. The programs allowed participants the space and time where they could experience sport, PA or recreation in an environment that was safe and comfortable. For example, when interpreters (facilitators) were available, the sessions were simplified, making it easier for the women to understand directions. Women were also able to do the activity in their own clothing with no requirements for a uniform or specific active wear. Another aspect that was mentioned by most women in each program was that the program being no cost, and O1 providing transport allowed them to be involved.

“*If O1 didn’t do this program I would not go anywhere.”*

*“See we come to the gym dressed like we are every day, I like this, I can’t wear tight things to exercise.”*

*“She [facilitator] helps us to understand what the man is saying for us to learn tennis, no understand English so hard to go by myself.”*

*“We come here by bus, it good they pick us up, we do the activity and then have food together.”*

*“It’s very good it’s free, too expensive for me to pay. I have lots of bills for my son and medicine.”*

### Barriers

Women in the focus groups were asked about some reasons why they and other people from the same community might not participate in sport, PA and recreation. The women also mentioned that they only participated in programs offered by O1 because they acknowledge that it was difficult to engage CALD women generally in mainstream programs. Indeed, while women in the groups tended to agree that sport, PA and recreation was important, they all went on to identify an extensive range of barriers that they believed shaped their experiences of sport, PA, and recreation activities among women from their cultures. The barriers that emerged from the study included: access barriers (language, facilities, transport), socio-cultural barriers (family responsibilities, culturally embedded priorities, gender roles, expectations), resource barriers (time, personal finances, lack of knowledge on services offered), interpersonal barriers (confidence, interpersonal networks) and physiological barriers (health, age).

#### Access barriers

One of the major barriers for CALD women was English language skills, especially for those who had not been in Australia for long, such as the Karenin and Karen women. Language proficiency shaped women's opportunities to find out about, access, and participate in activities or programs; poor English skills could leave women socially isolated and uninformed. English skills were seen as essential for participating in organised sport, PA and recreation classes or programs, as instruction is almost always in English. Some women mentioned that it made it difficult to mix with people outside of their cultural group because of this reason, limiting access to programs.

The availability of safe, accessible and culturally appropriate sport and recreation facilities were identified as important influences on participation. The woman wanted facilities to be safe, in good condition and easy to access. Some also mentioned more opportunities for women only programs and services offered at facilities during the day.

Another barrier discussed was transport. Having public transport, facilities close to home or organised transport by service providers was important to how the women could access sport, PA and recreation opportunities.

“*I don’t speak or read English, it is difficult for me to find things to do, I have to ask my children to explain but when I go to program, they are in English and my children are not there to explain to me what to do, very difficult, I am embarrassed so I don’t go to these programs.”*

*“I use a frame, getting to some programs is very difficult lots of steps and hard to walk inside.”*

*“I don’t drive and there is no public transport near me, it’s good that O1 organises a bus for us to get to the tennis program.”*

#### Socio-cultural barriers

Culturally embedded gender roles and family responsibilities were identified by women as reasons for the lack of leisure time and pervasive barriers to participant in sport, PA and recreation. Family responsibilities emerged as a major barrier in the study for all the women, especially for the ones that were carers of people with disabilities. Some women also described how in their culture, women were expected to be the caretakers of the home and children which limits their leisure time for themselves.

“*I need to do the housework, take children to school, cook, everything for my family. No time for activities for me.”*

*“In our culture, women are not encouraged to participate in sports, especially in mixed-gender environments. I wish there were more women-only fitness classes available.”*

*“When we were young, we used to look after the children and housework. When we grow up we have to look after the grandchildren.”*

*“Sport is not something that we do in our culture. Gardening keeps us active, housework.”*

*“As girls we never had the opportunity to do sport, it was for the boys.”*

#### Resource barriers

Many of the women explained how time was a barrier to participating in sport, PA and recreation. A common difficulty was a shortage of time, given the women's family, caring and work commitments. Some of them found that organised activities and programs in sport and recreation were not offered at times that were convenient to them. As well as time pressures, the women identified financial barriers to participating in sport and recreation. The cost was felt mostly by women who recently settled in Australia. Another resource barrier mentioned was the lack of knowledge of services available to them to participate in sport and recreation. Not being able to find, understand or identify options was identified in many of the discussions.

“*We don’t have time. That is the main problem. I have grandchildren, I have my daughter, by the time I help her and do my housework, the week is gone.”*

*“As a single mother, finding time for exercise is tough. I have to manage everything at home plus child with a disability. There’s hardly any time left for myself.”*

*“We can’t do fun things, we have so many other priorities in life that money is just hand to mouth, managing daily expenses, so we can’t even think about going to programs to exercise.”*

*“I can’t trust any other program, I don’t know who they are, where they are, what do they do. Its difficulty to find an activity I can do if I don’t know where they are.”*

#### Interpersonal barriers

CALD women's interpersonal networks and their confidence to engage in sport and recreation activities was a further barrier. Some women lacked confidence to participate as they didn’t know other people involved in the activities or did not know what to expect.

“*I would love to do more Yoga, but one of the things that stops me is, “what are other people going to think of me?” I always think I’m so terrible at Yoga and they are all so fantastic. I don’t want to make a fool of myself.”*

*“It’s nice to be together with others that understand me, I would not go to other programs without knowing other women.”*

*“This program run by [O1] started a dance class for women from my country. It feels like a little piece of home, and it’s fun!”*

*“The tennis class is not just about staying fit; it’s also where I meet my friends. We support each other.”*

*“I feel less isolated because I meet other mothers with this dance class. We’ve formed a little community group.”*

#### Physiological barriers

Physiological barriers to participation were also found to impede participation for some women in the study. These barriers related to health and age. Particularly older women's patterns of participation were affected by accidents and injuries as well as age-related problems like arthritis.

“*I have very bad pain in, my knees, I can’t do sport activities, it hurts too much.”*

*“I am too old now to do these [sport and recreation] activities all the time. I like to do them with this groups because I can do some and then stop and start again when I feel like it.”*

*“I started walking every day because my doctor told me it’s good for my heart. I’ve really noticed a difference in how I feel.”*

*“Since joining the swimming program, I’ve lost some weight, and my diabetes is easier to manage. It’s been a big change.”*

*“Yoga has been a great way for me to clear my mind. It’s my time to relieve stress and cope better with caring for my daughter[who has a disability].”*

## Discussion

Overall, this study found that there is limited research on the PA needs of carers from CALD backgrounds in Australia (29). However, many CALD communities have a strong tradition of family care, making support for family carers particularly crucial. One qualitative study in Australia of carers from Italian, Chinese, Spanish, and Arabic-speaking communities that identified barriers to using carer support services, such as unfamiliarity with formal services, a preference for family care, and a lack of ethno-specific services. This indicates a clear need for more ethno-specific services and enhanced cultural competency in mainstream services (29) such as O1 in this study.

On comparing the findings of this study to previous literature (17, 24) the primary enabler for CALD carers women's participation in the O1 program was the significant improvement in health and well-being. Participants reported increased physical competence, energy, motivation, and confidence. They gained valuable knowledge and understanding that emphasized the importance of being active in daily life. This improvement in health markers like blood pressure highlights the tangible health benefits of such programs which was also reported in previous studies with carers but not specific to CALD women carers.

An important finding in this study was that programs that impart life skills, such as swimming and self-defence, were particularly well-received and not previously reported in literature. These skills are not only essential for personal safety but also contribute to participants’ confidence and ability to engage more fully in their communities. Furthermore, social interactions and support networks formed during these activities were crucial for the participants. Many women appreciated the opportunity to build friendships and receive mutual support from other CALD carers, which significantly contributed to their continued participation. This sense of community and belonging is vital for mental and emotional well-being, especially for those who may feel isolated as found in Caperchione et al.'s (9), study. The supportive structure provided by O1, including the availability of interpreters, culturally appropriate environments, and organized transport, made it easier for CALD women carers to participate. The program's no-cost nature was also a significant enabler, as it removed financial barriers. One participant emphasized, “It’s very good it’s free, too expensive for me to pay. I have lots of bills for my son and medicine.” This support framework underscores the importance of accessibility and inclusivity in program design.

Language barriers were a significant challenge, especially for CALD women carers who had recently arrived in Australia from this study. Difficulty understanding English limited their ability to participate in and benefit from programs. As well, the availability of safe and accessible facilities was crucial. Transport issues further compounded these access barriers, as highlighted by Schaffler et al. (30) in their study. Cultural norms and family responsibilities often restricted leisure time for these women in the study. Traditional gender roles and expectations played a significant role in limiting their participation in sport and recreation. This indicates the need for programs to be flexible and considerate of these cultural and familial obligations.

A finding not dissimilar with other research for women (31) was time and financial constraints were common resource barriers. Many women had demanding schedules due to work, family and caregiving responsibilities, leaving little time for recreational activities. Financial limitations also hindered their ability to engage in paid programs. Furthermore, it is vital that women carers of CALD communities play an integral role in the co-development and co-delivery of these programs which allow for understanding the demands and needs they experience. Echoing the views of scholars like Caperchione et al. (9), Hartmann and Kwauk (32), and Jeanes et al. (33), understanding and integrating local cultural practices and community needs are essential for the success of any sport or recreation initiative. This research underscores the importance of designing programs that are co-created as much as possible, allowing for customization that caters to the unique benefits sought by all participants. For instance, a swimming program might adapt its sessions based on whether participants prioritize lifesaving skills, technique improvement, or simply the enjoyment and relaxation of swimming in a group setting. Programs need to be accessible, culturally sensitive, and supportive to maximize positive health outcomes and skill development while overcoming socio-cultural, resource, and physiological barriers. By understanding and mitigating these challenges, programs like those offered by O1 can significantly improve the quality of life and well-being of CALD women carers. Lastly, there should be a nuanced understanding of what “health” means to participants, incorporating methods that address mental well-being, such as relaxation techniques and stress reduction as well as physical health. Creating the right type of activities and environments that resonate with the participants’ definitions of health is crucial for programs focused on developmental rather than merely integrative outcomes.

Following on, this research identified that programs should have space for socialisation and relationship building. Programs should incorporate socialisation time before or after and advertise this element prior so that participants allow for the extra time that they devote to the program. Carers particularly often have limited time available to them to be involved in the program, hence important that they are aware of the opportunity to socialise, should they choose to participate. Some women spoke of the opportunity for O1 or groups to organise online catch ups between each sport or recreation program to motivate them to stay active beyond the scheduled programs.

Understanding the specific needs and constraints of CALD women carers is crucial for designing effective interventions that promote PA within this group. This study suggests that tailored programs that consider cultural preferences, provide linguistic support, and offer flexible scheduling options may improve engagement in PA. It is crucial to engage with community leaders and members in the planning phase to ensure that the initiatives are culturally relevant and sensitive. For example, in designing a PA program for Muslim women, incorporating preferences for single-sex sessions and providing culturally appropriate attire can make the difference in participation levels. Effective communication is key, providing information about PA and its benefits should not only be translated into the primary language of the target community but also delivered in a manner that is culturally resonant. This might mean using community channels, such as local leaders and religious centres, to disseminate information and engage participants. Understanding the daily rhythms and geographical nuances of CALD communities can aid in the successful implementation of PA programs. Programs may need to be scheduled around cultural events or religious observances and located in places that are both physically and socially accessible to women. In many CALD communities, religious institutions are central. Integrating PA programs into these settings can enhance acceptability and reach. For instance, churches, mosques, and temples can host exercise classes that align with religious events or timings, making it convenient and culturally acceptable for women to attend.

Despite these challenges, Australia's multicultural landscape presents significant opportunities for innovation and collaboration in promoting sport and PA participation among CALD women carers. By leveraging the strengths and resources of diverse communities and fostering partnerships across sectors, organizations can work towards creating more inclusive and equitable opportunities regardless of cultural background. This was a significant finding within this research, not reported previously for this cohort of CALD women carers.

## Conclusion

The current research explored the benefits for participants of a selection of PA programs which were designed and delivered specifically for women carers from a CALD background. These findings are important since government and non-government organisations such as O1 in Australia and internationally continue to invest in sport and recreation programs as well as programs to increase participation levels for CALD communities. Although this is one set of programs in one state of Australia, the value of the current research was to better understand the barriers and enablers for future program delivery.

While sport, PA, and recreation offer numerous benefits as found through this research study, disparities in participation rates persist among newly arrived people and individuals from CALD backgrounds in Australia (16). The influence of cultural norms on the PA patterns of CALD women is a complex issue that requires a nuanced understanding of the intersection between culture, gender, and health. Addressing these needs with culturally appropriate strategies can lead to improved health outcomes and a higher quality of life for CALD women carers (9). Addressing these disparities requires a comprehensive understanding of the social determinants influencing participation, as well as targeted strategies to promote inclusivity and equity. By attending to structural barriers, fostering cultural competency, and empowering CALD communities, stakeholders can work towards creating more accessible and inclusive sport or recreation environments. Aswell as ensuring there are effective interventions that respect and incorporate cultural values, providing pathways for CALD women carers to engage in PA while honouring their cultural identities.

Finally, through this study it found that organizations wanting to support CALD women carers need to look beyond the conventional approach of focussing on the actual delivery of the sport, PA, or recreation program but rather considerable efforts to empower and ensure that CALD community members play a significant role in co-development and co-delivery of any program to allow for sustainability of these programs from the onset.

## Data Availability

The raw data supporting the conclusions of this article will be made available by the authors, without undue reservation.

## References

[1] Agergaard S. Rethinking Sport and Integration: Developing a Transnational Perspective on Migrants and Descendants in Sports. London, UK: Routledge (2018).

[2] El Masri A Kolt GS George ES. Physical activity interventions among culturally and linguistically diverse populations: a systematic review. Ethn Health. (2022) 27(1):40–60. 10.1080/13557858.2019.1658183 31446773

[3] Bull FC Al-Ansari SS Biddle S Borodulin K Buman MP Cardon CC World health organization 2020 guidelines on physical activity and sedentary behaviour. Br J Sports Med. (2020) 54:1451–62. 10.1136/bjsports-2020-102955 33239350 PMC7719906

[4] Marquez DX Aguiñaga S Vásquez PM Conroy DE Erickson KI Hillman C A systematic review of physical activity and quality of life and well-being. Transl Behav Med. (2020) 10(5):1098–109. 10.1093/tbm/ibz198 33044541 PMC7752999

[5] Panza GA Taylor BA Thompson PD White CM Pescatello LS. Physical activity intensity and subjective well-being in healthy adults. J Health Psychol. (2019) 24(9):1257–67. 10.1177/1359105317691589 28810402

[6] An HY Chen W Wang CW Yang HF Huang WT Fan SY. The relationships between physical activity and life satisfaction and happiness among young, middle-aged, and older adults. Int J Environ Res Public Health. (2020) 17(13):4817. 10.3390/ijerph17134817 32635457 PMC7369812

[7] Suryadinata RV Wirjatmadi B Adriani M Lorensia A. Effect of age and weight on physical activity. J Public Health Res. (2020) 9(2):187–90. 10.4081/jphr.2020.1840 PMC7376490 32728579

[8] Paudel S Mishra GD Veitch J Mielke GI Hesketh KD. Examination of physical activity, organized sport, and sitting time among women and mothers from culturally and linguistically diverse backgrounds. J Phys Act Health. (2023) 21(3):229–37. 10.1123/jpah.2023-0061 38086350

[9] Caperchione CM Kolt GS Tennent R Mummery WK. Physical activity behaviours of culturally and linguistically diverse (CALD) women living in Australia: a qualitative study of socio-cultural influences. BMC Public Health. (2011) 11:1–10. 10.1186/1471-2458-11-26 21223595 PMC3091537

[10] Caperchione CM Kolt GS Mummery WK. Examining physical activity service provision to culturally and linguistically diverse (CALD) communities in Australia: a qualitative evaluation. PLoS One. (2013) 8(4):e627777. 10.1371/journal.pone.0062777 PMC3637443 23638145

[11] Janssens J Verweel P. The significance of sports clubs within multicultural society. On the accumulation of social capital by migrants in culturally ‘mixed’ and ’separate’ sports clubs. Eur J Sport Soc. (2014) 11(1):35–58. 10.1080/16138171.2014.11687932

[12] Tremblay M Bryan S Pérez C Ardern C Katzmarzyk P. Physical activity and immigrant status: evidence from the Canadian community health survey. Can J Public Health. (2006) 97(4):277–82. 10.1007/BF03405603 16967745 PMC6976030

[13] Maxwell H Stronach M. Developing sport for culturally and linguistically diverse women and girls. In: Developing Sport for Women and Girls. London, UK: Routledge (2020). p. 83–94.

[14] Whitley MA Coble C Jewell GS. Evaluation of a sport-based youth development programme for refugees. Leisure/loisir. (2016) 40(2):175–99. 10.1080/14927713.2016.1219966

[15] Australian Bureau of Statistics. Australia’s Population by Country of Birth. Canberra, Australia: ABS (2021). Available online at: https://www.abs.gov.au/statistics/people/population/australias-population-country-birth/2021 (Accessed January 15, 2024).

[16] Australian Sport Commission. Clearinghouse for Sport, Cultural Diversity in Sport (2018). Available online at: https://www.clearinghouseforsport.gov.au/data/assets/pdf_file/0004/865201/Cultural_Diversity_and_the_Role_of_Sport.pdf (Accessed January 15, 2024).

[17] Temple J Dow B Kosowicz L. Carers of older Australians: unmet support needs and carer well-being. Aust J Prim Health. (2021) 27(3):178–85. 10.1071/PY20161 33993903

[18] Poon AWC Lee JS. Carers of people with mental illness from culturally and linguistically diverse communities. Aust Soc Work. (2019) 72(3):312–24. 10.1080/0312407X.2019.1604300

[19] Gilbert AS Antoniades J Croy S Thodis A Adams J Goeman D The experience of structural burden for culturally and linguistically diverse family carers of people living with dementia in Australia. Health Soc Care Community. (2022) 30(6):e4492–503. 10.1111/hsc.13853 35599431 PMC10083988

[20] Walker R Belperio I Gordon S Hutchinson C Rillotta F. Caring for a family member with intellectual disability into old age: applying the sociocultural stress and coping model to Italian and Greek migrants in Australia. J Appl Res Intellect Disabil. (2020) 33(5):887–97. 10.1111/jar.12710 32072718

[21] Lee DCA Haines TP Callisaya ML Hill KD. A scalable program for improving physical activity in older people with dementia including culturally and linguistically diverse (CALD) groups who receive home support: a feasibility study. Int J Environ Res Public Health. (2023) 20(4):3662. 10.3390/ijerph20043662 36834355 PMC9959901

[22] Poon AWC Cassaniti M Sapucci M Ow R. Wellbeing and experiences of Chinese and Vietnamese carers of people with mental illness in Australia. Transcult Psychiatry. (2021) 58(3):351–64. 10.1177/1363461520952629 32969331

[23] O’Driscoll T Banting LK Borkoles E Eime R Polman R. A systematic literature review of sport and physical activity participation in culturally and linguistically diverse (CALD) migrant populations. J Immigr Minor Health. (2014) 16:515–30. 10.1007/s10903-013-9857-x 23771744

[24] Spaaij R Luguetti C McDonald B McLachlan F. Enhancing social inclusion in sport: dynamics of action research in super-diverse contexts. Int Rev Sociol Sport. (2023) 58(4):625–46. 10.1177/10126902221140462

[25] Wohler Y Dantas JA. Barriers accessing mental health services among culturally and linguistically diverse (CALD) immigrant women in Australia: policy implications. J Immigr Minor Health. (2017) 19:697–701. 10.1007/s10903-016-0402-6 27002625

[26] Khawaja NG Moisuc O Ramirez E. Developing an acculturation and resilience scale for use with culturally and linguistically diverse populations. Aust Psychol. (2014) 49(3):171–80. 10.1111/ap.12052

[27] Braun V Clarke V. Conceptual and design thinking for thematic analysis. Qual Psychol. (2022) 9(1):3. 10.1037/qup0000196

[28] Braun V Clarke V. Reflecting on reflexive thematic analysis. Qual Res Sport Exerc Health. (2019) 11(4):589–97. 10.1080/2159676X.2019.1628806

[29] Shanley C Boughtwood D Adams J Santalucia Y Kyriazopoulos H Pond D A qualitative study into the use of formal services for dementia by carers from culturally and linguistically diverse (CALD) communities. BMC Health Serv Res. (2012) 12:1–11. 10.1186/1472-6963-12-354 23043332 PMC3523018

[30] Schaffler JL Tremblay S Laizner AM Lambert S. Developing education materials for caregivers of culturally and linguistically diverse patients: insights from a qualitative analysis of caregivers’ needs, access and understanding of information. Health Expect. (2019) 22(3):444–56. 10.1111/hex.12867 30767349 PMC6543161

[31] Farrugia T Hewitt A Bourke-Taylor H Joosten AV. The impact of carer status on participation in healthy activity and self-reported health among Australian women over 50 years. Aust Occup Ther J. (2019) 66(1):23–32. 10.1111/1440-1630.12491 29920685

[32] Hartmann D Kwauk C. Sport and development: an overview, critique, and reconstruction. J Sport Soc Issues. (2011) 35(3):284–305. 10.1177/0193723511416986

[33] Jeanes R O’Connor J Alfrey L. Sport and the resettlement of young people from refugee backgrounds in Australia. J Sport Soc Issues. (2015) 39(6):480–500. 10.1177/0193723514558929

